# The modified Manchester Fothergill procedure compared with vaginal hysterectomy with low uterosacral ligament suspension in patients with pelvic organ prolapse: long-term outcome

**DOI:** 10.1007/s00192-022-05240-3

**Published:** 2022-06-02

**Authors:** Rosa A. Enklaar, Femke M. F. M. Knapen, Sascha F. M. Schulten, Liesbeth A. D. M. van Osch, Sanne A. L. van Leijsen, Ed T. C. M. Gondrie, Mirjam Weemhoff

**Affiliations:** 1 Department of Obstetrics and Gynecology, Zuyderland Medical Center, Heerlen, The Netherlands; 2 Radboud Institute for Health Sciences, Department of Obstetrics and Gynecology, Radboud University Medical Center, Nijmegen, The Netherlands; 3 Department of Health Promotion/CAPHRI, Maastricht University, Maastricht, the Netherlands; 4 Department of Clinical Genetics, Maastricht University Medical Centre +, Maastricht, The Netherlands; 5 Department of Obstetrics & Gynecology, Maxima Medical Centre, Veldhoven, The Netherlands

**Keywords:** Pelvic organ prolapse, Manchester Fothergill, Vaginal hysterectomy, Long-term outcome

## Abstract

**Introduction and hypothesis:**

The objective of this study was to compare the long-term outcome between vaginal hysterectomy with low uterosacral ligament suspension (VH) and the modified Manchester Fothergill procedure (MF) as surgical treatment in patients with pelvic organ prolapse (POP). We hypothesize that MF is non-inferior to VH in the long term.

**Methods:**

In this single-center retrospective cohort study patients who underwent MF or VH for primary apical compartment prolapse between 2003 and 2009 were eligible for inclusion. The primary outcome was subjective recurrence of POP. Secondary outcomes included number and type of reinterventions, time to reintervention and the degree of complaints.

**Results:**

One hundred sixty of 398 patients (53 MF, 107 VH) returned the questionnaires (40%). The mean follow-up was 12.97 years for MF and 13.24 years for VH (*p* = 0.38). There were similar rates of subjective POP recurrence (51% in both groups). The reintervention rate in the MF group was higher but reached no statistical significance [19/53 (36%) versus 29/107 (27%), *p* = 0.26]. Kaplan-Meier curve showed no statistically significant difference in risk of reintervention after MF at the maximum follow-up of 16.5 years [HR 1.830 (95% CI 0.934–3.586), *p* = 0.08]. The mean time to reintervention was 3 years shorter in the MF group (*p* = 0.03)*.*

**Conclusions:**

The subjective recurrence after MF is similar to VH in treatment of POP at the long term. MF appears to be non-inferior to VH when comparing the risk of reintervention. However, the small sample size precludes a definitive conclusion of non-inferiority, and future studies are needed.

**Supplementary Information:**

The online version contains supplementary material available at 10.1007/s00192-022-05240-3.

## Introduction

Pelvic organ prolapse (POP) is a common gynecological disorder in aging women, affecting 40% of women > 45 years old [1]. A woman’s lifetime risk of surgery for POP by the age of 80 is 11%, and nearly 1/3 of these women will undergo a re-operation because of a recurrence [2]. Not all women with POP have complaints. However, when experiencing complaints the quality of life can be reduced significantly [3]. Although POP is common, there is no consensus on the best surgical treatment of an apical prolapse. This is resulting in wide practice pattern variation [4, 5].

The vaginal hysterectomy with low uterosacral ligament suspension (VH) is traditionally one of the most widely used surgeries to treat apical prolapse, but it also has its drawbacks [6]. Vaginal hysterectomy is associated with longer operation time, more intra- and postoperative bleeding and a longer recovery [7, 8]. The absence of a roof tile construction of the pelvic floor after removal of the uterus weakens the pelvic support system after the intervention [9]. Uterus-preserving procedures for POP, such as Manchester Fothergill (MF), have been gaining popularity over the last years. Some of the benefits of uterus-preserving surgery are that it is a less invasive procedure with fewer perioperative complications, has a shorter operation time and hospitalization and therefore also has a lower cost compared to the VH [7]. These benefits together with recently published evidence [7, 10] of a good outcome regarding the chance of a recurrent prolapse after uterus-preserving surgeries have caused an increase in the number of performed modified Manchester Fothergill operations (MF) [11].

Several studies showed that women have a growing preference towards uterus-preserving surgery [12, 13]. A recent publication showed a significant change in the approach to POP in The Netherlands; the number of VHs decreased from 2560 (26.8% of POP surgery) to 1213 (19.0% of POP surgery) with a relative difference of -33.6% (95% CI -38.9 to 27.7; *p* = 0.0001) from 2011 to 2017. Furthermore, a decrease in practice pattern variation for POP surgery and for VH has been described [5]. Different factors might influence the practice variation such as patient characteristics, the preference of gynecologists, and the patients’ preference.

Despite MF being executed for POP for more than a century, little is known about the long-term results of this procedure. To date, only a few studies on the results of the MF procedure with a longer term follow-up have been published [7, 14, 15]. Studies comparing MF with VH show conflicting results, with some studies showing lower recurrence rates after MF [7, 15] and others indicating that VH has similar results as MF [14]. The maximum follow-up time in these studies was 6.5 years.

The aim of this study is therefore to compare the long-term follow-up outcome after a minimal follow-up period of 10 years of the VH and MF procedure regarding subjective recurrence rates, reintervention rates and time to reintervention. We hypothesize that the MF procedure is non-inferior to VH when compared after a minimal follow-up period of 10 years.

## Materials and methods

### Study population

This study is a retrospective, single-center study. The study population consists of all patients who underwent a modified MF or VH with low uterosacral ligament suspension for primary apical compartment prolapse between 2003 and 2009 in the Orbis Medical Center in Sittard, The Netherlands. Each patient who received MF or VH with low uterosacral ligament suspension for apical prolapse was eligible for inclusion; a concomitant anterior and/or posterior colporrhaphy was allowed. One surgeon mainly performed the MF; the VH was performed by three surgeons. All four surgeons in the study were experienced in performing benign gynecological surgery including prolapse surgery. Exclusion criteria for this study were death and inability to complete the questionnaire, e.g., because of dementia. Vaginal hysterectomy for other reasons than POP (such as cervical or endometrial carcinoma or benign conditions such as bleeding disorders) was registered and excluded for this study. Patient characteristics and preoperative Pelvic Organ Prolapse-Quantification (POP-Q) stages were collected from the electronic patient files and using questionnaires. In some cases preoperative POP stages were determined using Baden-Walker quantification instead of POP-Q. In these cases, the measurements were recoded per compartment to a POP-Q stage. Patients were not matched.

### Recruitment and study procedure

All patients were informed by letter about this study. The electronic patient file was checked for address details and to verify that patients had not passed away. The informative letter included information concerning the study procedure, an informed consent form and a questionnaire (Appendix A). Patients were asked to confirm or refuse their participation in the study. When patients agreed to participate in the study, they were asked to return the informed consent and completed questionnaire. If patients did not respond after 2 to 3 weeks, they were approached by telephone. Ethical approval was required and granted by the Medical Ethics Committee of Zuyderland (Study ID Z202011).

### Surgical procedures

#### Modified Manchester Fothergill procedure

The Modified Manchester Fothergill procedure is a uterus-preserving operation. First, the cervix is held by a tenaculum forceps and circumcised. This is followed by dissection of 2–3 cm of the vaginal wall to cover the residual of the cervix at the end of the operation. The bladder is dissected off the cervix over 2 to 3 cm. The peritoneal cavity is not opened. The surgeon identifies the uterosacral ligaments (USL), at the lateral posterior side of the cervix. The ligaments are ligated with three to four Vicryl sutures. Between the sutures there is 0.5 cm distance. The most cranial uterosacral ligament suture enters in the posterior fornix but is not knotted at this point yet. At the anterior site the cardinal ligaments are ligated with one suture. An anterior colporrhaphy is performed if necessary. Then, the cervix is amputated over 1 to 2.5 cm, depending on the cervical elongation. The cervical canal is dilated to ensure uterine drainage. A posterior and anterior Sturmdorf suture is used to construct a neoportio by covering the amputated cervix with vaginal mucosa. The most cranial uterosacral ligament in the fornix posterior is knotted. If necessary, a posterior colporrhaphy is performed afterwards.

#### Vaginal hysterectomy procedure with low uterosacral ligament suspension

The vaginal hysterectomy is the removal of the uterus through the vagina. First, the cervix is held by a tenaculum forceps and circumcised. Anteriorly, the bladder is dissected from the cervix and vagina, and the anterior peritoneum is opened. Posteriorly to the cervix, the pouch of Douglas is opened as well. Then, the uterosacral ligaments are identified, clamped and ligated with a Vicryl suture. The sutures are left long. The uterus is further detached from the surrounding structures, such as the cardinal ligaments with the uterine vessels, tubo-ovarian ligaments and round ligaments, after which the uterus is removed. The surgeon will proceed with closing and fixating the vaginal vault. To prevent future POP of the vaginal vault, a low uterosacral ligament suspension is performed by attaching the uterosacral ligaments with sutures to the vaginal vault. Last, the vaginal vault is closed with interrupted sutures. If necessary, an anterior and/or posterior colporrhaphy is performed afterwards.

### Outcome measurements

The primary outcome was occurrence of a subjective recurrence of POP. Subjective recurrence was defined as feeling or seeing a bulge from the vagina [16, 17]. The secondary outcome was reintervention and included physiotherapy because of POP symptoms, a pessary or a re-operation. If patients had undergone multiple reinterventions, the most invasive intervention was included in the analysis, in the sequence: re-operation > pessary > physiotherapy. Furthermore, the secondary outcomes pertained to the degree of complaints in women after MF and after VH. These complaints were related to micturition, defecation and the influence of symptoms on the daily functioning of the patient, regardless of the occurrence of a possible recurrent POP. Data of a subjective recurrence, reinterventions and complaints were obtained by validated health-related and disease-specific questionnaires (Appendix A). This set of questionnaires, compiled by the Dutch Society for Urogynecology, includes the Incontinence Impact Questionnaire (IIQ), Urogenital Distress Inventory (UDI) and Defecation Distress Inventory (DDI), which have been validated for the Dutch language [14, 18–21]. These questionnaires consist of a 4-point Likert scale where patients can score various symptoms ranging from no complaints to serious complaints and score for different subdomains [18, 19]. These scores range between 0 (no symptoms or no bothersome symptoms for UDI and DDI, best quality of life for IIQ) to 100 (most bothersome for UDI and DDI, worst quality of life for IIQ). The subscores of the UDI were divided into overactive bladder, urinary incontinence, obstructive micturition, pain and genital prolapse*. The UDI questionnaire includes the following questions: “Do you experience a sensation of bulging or protrusion from the vagina?”* and *“Do you have a bulge or something falling out that you can see in the vagina?”* The number of recurrent urinary tract infections was retrieved by using one specific question in the questionnaire (‘How often did you suffer from a urinary tract infection in the last year?’). The subscores of the DDI were divided into constipation, obstructive defecation, pain, and fecal and flatus incontinence. The IIQ covers five domains, i.e., physical functioning, mobility, social functioning, embarrassment and emotional functioning.

We collected data of all patients who underwent a MF procedure to estimate the number of cervical stenoses, endometrial cancer and uterus-removing procedures after the initial MF procedure. With uterus-preserving surgery gaining more popularity for the surgical treatment of apical prolapse, there is a worldwide interest in this specific disadvantage after MF.

### Statistical analysis

Data collection and analysis were performed by the same investigators. The mean and standard deviation (SD) are reported for continuous data. For discrete data, the count and percentage are reported. For comparison of baseline variables between groups, we used the independent *t*-test for normally distributed numerical values, Mann-Whitney U test for skewed distributed numerical values and chi-square or Fisher’s exact test for categorical variables. We performed time-to-event (survival) analysis using Kaplan-Meier curves to estimate the differences and incidence during follow-up. Censored cases in the Kaplan-Meier curves represent patients who completed the follow-up at the moment they returned the questionnaires. The at-risk population with the survival analysis was presented per year of follow-up. The recurrence rate was also presented in number of events per patient-years and was calculated by dividing the number of events by the total amount of person-time at risk for both operation techniques. To improve comparability across studies, the recurrence rate was displayed as the number of events per 100 patient-years.

Log-rank (Mantel-Cox) was applied for testing of equality of the survival distributions. Hazard ratio (HR) and 95% confidence interval (95% CI) were calculated using Cox regression and were adjusted for BMI, age, anterior vaginal wall prolapse, posterior vaginal wall prolapse stage > 2 and POP-Q stage > 3 in any compartment. This number of variables is within the range of 10% of events. Multivariable logistic regression analysis was performed to test for confounding variables for both subjective recurrence and reintervention. The following variables were included in the logistic regression analysis for subjective recurrence as they might be confounding variables [22]: body mass index (BMI), age, birthweight of a child > 4 kg, presence of anterior vaginal wall prolapse and posterior vaginal wall prolapse stage > 2 and preoperative higher POP-Q stage > 3 in any compartment. A logistic regression analysis was performed to test the relation between the age of the participants and score of the urinary incontinence domain of the UDI questionnaire.

A *p*-value of 0.05 was considered significant for all comparisons. All data were entered and analyzed in SPSS 25.0 for Windows.

## Results

**Fig. 1:**
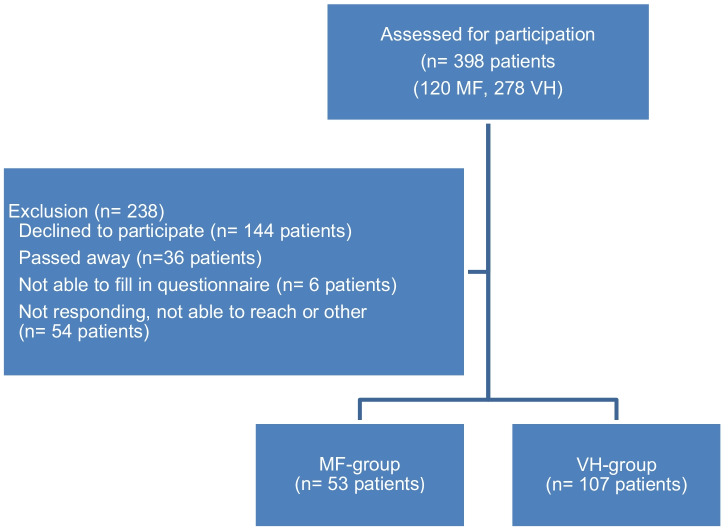
Flowchart participants and reasons for exclusion (MF: modified Manchester Fothergill procedure, VH: vaginal hysterectomy with low uterosacral ligament suspension)

For this study, 398 patients were eligible for participation and received an invitation: 120 patients who underwent a MF procedure and 278 patients who had had a VH. In total, 160 patients returned the questionnaire (40%); 53 respondents were treated with the MF and 107 with VH. This resulted in a response rate of 44% and 38%, respectively (Fig. 1). Table 1 shows the baseline characteristics of the 160 patients. We found a statistically significant younger age in the VH group compared to the MF group (51 ± 9 vs. 57 ± 9 years, *p* = 0.001) (Table 1). A statistically significant majority of patients who underwent MF had concomitant anterior colporrhaphy compared with patients who underwent VH for apical prolapse (30 (56%) vs. 39 (37%), *p* = 0.02).

**Table 1:** Baseline characteristics

	MF (*n* = 53)	VH (*n* = 107)	Significance (*p*)
Age at surgery (years) (mean ± SD)	57 ± 9	51 ± 9	0.001^a^
BMI^e^ (kg/m^2^) (mean ± SD)	25.8 ± 3.9	26.7 ± 4.5	0.46^b^
Parity			
None ≥ 1	0 (0%) 53 (100%)	1 (1%) 106 (99%)	1.00^d^
Vaginal deliveries			
None ≥ 1	0 (0%) 53 (100%)	1 (1%) 106 (99%)	1.00^d^
Cesarean sections			
None ≥ 1	51 (96%) 2 (4%)	105 (98%) 2 (2%)	0.60^d^
Birthweight child > 4000 g	13 (25%)	38 (36%)	0.16^c^
Preoperative POPQ stage (stage > 2)			
Apical compartment Anterior compartment Posterior compartment	41 (77%) 46 (86%) 15 (28%)	78 (72%) 89 (83%) 45 (42%)	0.96^c^ 0.31^c^ 0.03^c^
Preoperative POPQ stage 3 or 4			
In any compartment	40/51 (78%)	74/103 (72%)	0.38^c^
Concomitant surgery			
No concomitant surgery Anterior colporrhaphy Posterior colporrhaphy Anterior and posterior colporrhaphy	3 (6%) 30 (56%) 3 (6%) 17 (32%)	12 (11%) 39 (37%) 15 (14%) 41 (38%)	0.39^c^ 0.02^c^ 0.12 ^c^ 0.44 ^c^
Preoperative mid-urethral sling surgery (TVT & TOT)	1 (2%)	3 (3%)	1.00^d^

Data are mean (standard deviation) or numbers (percent). Numbers not adjusted for missing data

^a^Independent samples *t*-test, ^b^Mann-Whitney U test, ^c^chi-square test, ^d^Fisher’s exact test, ^e^body mass index

Table 2 shows the subjective recurrence rate, reintervention rate and details concerning follow-up duration. We found no differences in the occurrence of a subjective POP recurrence between both groups. With a mean follow-up of 13 years (MF = 12.97 vs. VH = 13.24; *p* = 0.38), we found a subjective recurrence rate of 51% for the MF as well as the VH group. The multivariable logistic regression analysis for subjective recurrence showed no significant confounding factors and no difference for operation type [odds ratio (OR) 0.962 (95% CI 0.456–2.114, *p* = 0.96)].

**Table 2:** Subjective recurrence, reintervention rate and details concerning follow-up

	MF (*p* = 53)	VH (*p* = 107)	Significance (*n*)
Subjective POP recurrence	27 (51%)	55 (51%)	0.96^c^
Reintervention Physiotherapy for POP Pessary Surgery	19 (36%) 3 (5%) 4 (8%) 12 (23%)	29 (27%) 6 (6%) 10 (9%) 13 (12%)	0.26^c^ 1.00^d^ 1.00^d^ 0.09^c^
Time to reintervention (years)(mean ± SD) Time to re-operation (years)(mean ± SD) Follow-up duration (years)(mean ± SD)	5.39 ± 4.26 5.83 ± 4.57 12.97 ± 1.65	8.21 ± 3.99 7.54 ± 4.58 13.24 ± 1.63	0.03^b^ 0.36^a^ 0.38^b^

Data are mean (standard deviation) of numbers (percent)

^a^Independent samples *t*-test, ^b^Mann-Whitney U test, ^c^Chi-square test, ^d^Fisher’s exacttest

**Fig. 2:**
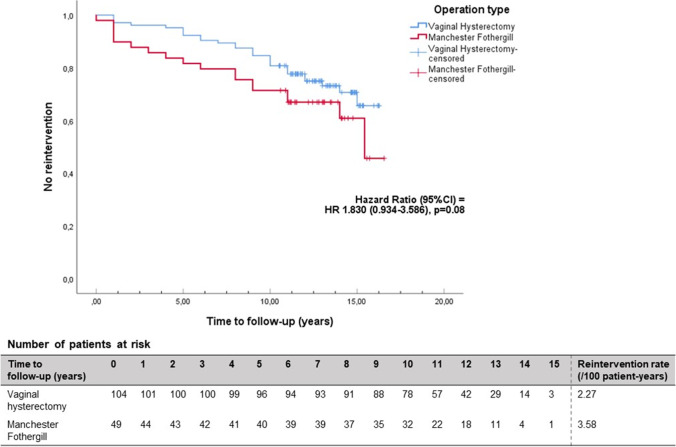
Kaplan-Meier curve showing reintervention rates for recurrent prolapse for both operations. The blue line represents the vaginal hysterectomy with low uterosacral ligament suspension and the red line the modified Manchester Fothergill procedure. The number of patients at risk is presented per year of follow-up. The reintervention rate in patient-years is calculated as the number of events divided by the amount of person-time at risk and converted to number of patients with reintervention per 100 patient-years

The reintervention rate was 36% (19/53) in the MF group versus 27% (29/107) in the VH group. The difference was not statistically significant (*p* = 0.26). The multivariable logistic regression analysis for reintervention showed no statistically significant confounders and no difference for operation type either [OR 1.914 (95% CI 0.836–4.379, *p* = 0.12] (Table 3). The Kaplan-Meier curve showed no statistically significant difference in the risk of reintervention at the maximum follow-up of 16.5 years between the two procedures [hazard ratio (HR) 1.83 (95% CI 0.934–3.586, *p* = 0.08)]. Because several censored cases occurred after 10 years, we performed a sensitivity analysis with a cut-off point of 10 years follow-up. This showed no differences [HR 1.813 (95% CI 0.935–3.514), *p* = 0.07]. Additionally to the Kaplan-Meier curve (Fig. 2), the recurrence rate in number of events per patient-year and the population at-risk is presented per year of follow-up for both MF and VH.

**Table 3:** Logistic regression analysis (univariable and multivariable) for subjective recurrence and reintervention after modified Manchester Fothergill versus vaginal hysterectomy with low uterosacral ligament suspension

	Univariable		Multivariable	
	OR (95% CI)	Significance (*p*)	OR (95% CI)	Significance (*p*)
**Subjective recurrence**				
Operation type (MF versus VH)	0.982 (0.508–1.897)	0.96	0.962 (0.456–2.114)	0.96
BMI (kg/m^2^)^a^	1.001 (0.931–1.076)	0.98	0.989 (0.913–1.071)	0.79
Age (years)	0.968 (0.936–1.000)	0.045	0.967 (0.931–1.005)	0.09
Birthweight child > 4 kg	0.696 (0.357–1.358)	0.29	0.746 (0.348–1.599)	0.45
POP-Q (st > 2)				
Anterior compartment Posterior compartment	1.585 (0.570–4.409) 1.116 (0.574–2.170)	0.38 0.75	2.160 (0.700–6.663) 1.227 (0.570–2.639)	0.18 0.60
POP-Q stage 3 or 4 in any compartment	0.485 (0.230–1.022)	0.06	0.499 (0.223–1.118)	0.09
**Reintervention**^**b**^				
Operation type (MF versus VH)	1.503 (0.743–3.042)	0.26	1.914 (0.836–4.379)	0.12
BMI (kg/m^2^)^a^	0.975 (0.899–1.057)	0.54	0.961 (0.880–1.050)	0.38
Age (years)	0.964 (0.930–1.00)	0.05	0.967 (0.928–1.007)	0.10
POP-Q (st > 2)				
Anterior compartment Posterior compartment	0.800 (0.277–2.309) 1.968 (0.963–4.025)	0.68 0.06	1.177 (0.376–3.686) 2.102 (0.933–4.739)	0.86 0.07
POP-Q stage 3 or 4 in any compartment	0.650 (0.304–1.390)	0.27	0.580 (0.252–1.335)	0.20

^a^BMI: body mass index. ^b^Logistic regression analysis for reintervention adjusted for the same variables as used in Cox regression analysis (= 10% of number of events)

The mean time to undergo a reintervention of any type for recurrent POP was 3 years shorter in the MF group as compared to the VH group: [5.39 ± 4.26 vs. 8.21 ± 3.99 years (*p* = 0.03)]. The mean time to re-operation was 1.7 years shorter in the MF group compared to the VH group (5.83 ± 4.57 years vs. 7.54 ± 4.58 years, *p* = 0.36). The latter was not statistically significant (Table 2).

Table 4 compares the degree of complaints related to micturition, defecation and functional outcome between both groups by means of follow-up scores of the UDI, DDI and IIQ. No statistically significant differences in the DDI and IIQ domain scores were found. In the UDI questionnaire, a significantly higher median score was found in the urinary incontinence domain for the MF group (*p* = 0.03), which means that patients reported more complaints of urinary incontinence after MF than after VH.

**Table 4:** Functional outcome: follow-up scores of the Urinary Distress Inventory (UDI), Defecation Distress Inventory (DDI) and Incontinence Impact Questionnaire (IIQ)

	MF (*n* = 53)	VH (*n* = 107)	Significance (*p*) ^a^
**UDI domain scores**			
Overactive bladder	22 (0–44)	11 (0–33)	0.27
Urinary incontinence	33 (8–33)	17 (0–33)	0.03
Obstructive micturition	0 (0–33)	0 (0–33)	0.46
Pain	0 (0–33)	0 (0–33)	0.71
Genital prolapse	0 (0–17)	0 (0–17)	0.43
Recurrent urinary tract infection	0 (0–33)	0 (0–33)	0.52
**DDI domain scores**			
Constipation	0 (0–17)	0 (0–17)	0.74
Obstructive defecation	0 (0–13)	0 (0–8)	0.30
Pain	0 (0–0)	0 (0–0)	0.12
Fecal incontinence	0 (0–17)	0 (0–17)	0.34
Flatus	33 (0–67)	33 (0–33)	0.25
**IIQ domain score**			
Physical functioning	0 (0–17)	0 (0–0)	0.29
Mobility	0 (0–22)	0 (0–22)	0.44
Social functioning	0 (0–11)	0 (0–0)	0.27
Embarrassment	0 (0–17)	0 (0–17)	0.35
Emotional health	0 (0–22)	0 (0–11)	0.13

Data are median (interquartile ranges); ^a^Mann-Whitney U test

Because baseline characteristics showed a younger age in the VH group compared to the MF group, a logistic regression analysis was performed. In this analysis age appeared not to be a significant confounder [OR 1.998 (95% CI 0.926–4.315), *p* = 0.08].

### Negative effects after Manchester procedure

Of the initial study population of patients who underwent MF (*n* = 120), only one patient received a uterus-removing procedure because of endometrial hyperplasia with cellular atypia. After the MF procedure only four cases of cervical stenosis were reported (e.g., slightly more difficult hysteroscopy procedure or IUD insertion). No hematometra was described. One patient had a pyometrium postoperatively, which was easily cured by dilating the cervical canal.

## Discussion

This study compared the long-term outcome of the modified Manchester Fothergill procedure (MF) with vaginal hysterectomy with low uterosacral ligament suspension (VH) in the treatment of apical compartment prolapse. We found that at the long term the subjective recurrence after MF procedure is similar to VH in treatment of pelvic organ prolapse (51% for both procedures). There was no significant difference in the risk of reintervention between the two procedures.

In the baseline characteristics, we found a significantly higher age in the MF group compared to the VH group. Age appeared to be a significant confounding factor in the univariable logistic regression analysis for subjective recurrence. However, in the multivariable analysis age was not statistically significant (*p* = 0.09). It is possible that for younger women the advantage of not having menstrual bleeding anymore could have influenced their preference for VH compared to older women, which might explain the age difference between the two groups. In addition to the age difference between the two groups, we found that more women suffered from a concomitant posterior compartment prolapse (POP-Q stage > 2) preoperatively in the VH group. In the multivariable analysis, this difference was also not statistically significant (*p* = 0.08). A Dutch review on risk factors for (recurrent) POP by Vergeldt et al. found inconsistent results for age as a risk factor for recurrent POP [22].

Denman et al. determined the 10-year risk of repeat surgery for surgically treated POP and urine incontinence (UI) and proved the overall risk for reoperation to be at 17% after 10 years [23]. Their study included all operations for both POP and UI (including retropubic urethropexies for urinary incontinence, sacrospinous hysteropexies, and vaginal and abdominal hysterectomies), as in our study we calculated the reintervention rate (including physiotherapy, pessary treatment and surgery) for POP recurrence after MF and VH. When we consider only the reoperation rate for recurrent POP, we found a reoperation rate of 23% for the MF group and 12% for the patients who underwent VH after a mean follow-up period of 13 years. This was not statistically significant (*p* = 0.09), but might be clinically relevant for patients. The mean reoperation rate of all patients in our study together (15%) is comparable with the results from Denman et al. The recurrence rate in the study by Thys et al. was 19% in the MF group and 18% in the VH group (*p* = 0.86) [14]. This lower recurrence rate in Thys’ study compared to our study may be due to a shorter follow-up time and the fact that they performed physical examination and therefore could distinguish between a recurrence POP and a de novo POP in the non-operated compartment in patients. Withagen et al. showed that 17% of patients develop a de novo POP in another non-operated compartment after conventional prolapse surgery within a year [24]. Ünlübilgin et al. compared MF and VH in a prospective, randomized controlled trial. They found that 2.0% in MF and 6.6% in VH had repeat surgery for prolapse recurrence after a mean follow-up of 61 months [15]. Meriwether et al. compared the MF with VH in a systematic review and meta-analysis and found no differences in risk of re-operation between the two operations (RR 0.42, 95% CI 0.15–1.15, *p* = 0.09) [25]. In contrast, a retrospective Danish study by Tolstrup et al., based on data from four Danish databases, was the only study that found a higher recurrence rate (defined as POP in previously operated area in any compartment) of 18.3% after a VH compared to 7.8% after MF. The follow-up duration was 51 and 48 months, respectively [7].

We collected data of all patients who underwent a MF procedure to estimate the number of endometrial cancers. We found only 1 out of 120 patients who underwent a uterus-removing procedure after MF because of abnormal endometrial cells. This is consistent with results of a retrospective cohort of a Danish healthcare database study by Husby et al., who found no increased risk of endometrial cancer and a low incidence of 1% [26].

On the UDI questionnaire, the MF group reported significantly higher scores on the urinary incontinence domain. This result has not been found in other literature. When interpreting the median scores for urinary incontinence [MF 33 (IQR 8–33) vs. VH (17 (IQR 0–33) on a 0–100 scale, *p* = 0.03], the median scores for patients with VH fall below the cutoff score of 33.33, which was determined to distinguish between symptomatic and asymptomatic women [21, 27]. We found no significant differences in the DDI and IIQ domain scores between both groups. In future prospective studies, the incidence of de novo incontinence after uterus-preserving techniques is an important factor to study, since this might influence the patients’ preference.

Our study includes some strengths and limitations. One of the limitations is the retrospective character of the study and therefore not having standardized questionnaires on symptoms preoperatively. However, a cohort with a long follow-up duration as in our current study can provide useful data.

Second, in this current study we collected solely questionnaires. Therefore, no distinction can be made between a de novo POP in a non-operated compartment and a recurrent POP because patients have not undergone a new POP-Q. In fact, the number of anatomical recurrent POPs in our study may actually be even higher than the subjective recurrence rate found because most (60.9%) recurrences are asymptomatic regarding prolapse sensation [18]. However, we believe that subjective symptoms of prolapse are leading in the request for a reintervention and it is not likely that patients receive a reintervention when they do not have any recurrence. We do not expect this limitation to have an impact on our conclusion regarding differences between the two surgical techniques.

Third, the result may be influenced by the fact that the MF procedure was mainly performed by one surgeon and the VH by more surgeons. However, all surgeons had a lot of experience in the performed procedures, so it is unlikely that a possible learning curve might have influenced the results.

Fourth, a limitation is the small size of the study population. It is possible that the difference between the reintervention rates of the two procedures did not reach statistical significance because of the small sample size. With a larger study population, this could possibly have been statistically significant. Even though the sample size is small, we think our results on the long-term outcome of the MF procedure contribute to the available evidence because the literature on this topic is scarce. The MF procedure has been described since the late 1800s, but in the literature only a few articles have been published concerning the long-term outcome of this procedure.

Finally, the loss to follow-up was high because of the long follow-up and the high age of the study population. Additionally, since this study was enrolled during COVID-19, it is possible the epidemic influenced the participation rate. The study population may not be completely representative for the overall population of women who have had POP surgery in the past, because a possible reason for participation was the presence of complaints. Some of the patients indicated that they did not want to participate because they no longer had any complaints. This may have led to an overestimation of the number of recurrences and the degree of complaints. On the other hand, patients who were frustrated by recurrent symptoms or who had sought treatment elsewhere could also have declined to participate. This potential representation and participation bias would, however, apply to the total study population and does not explain a difference between the two studied groups. Because the outcome reintervention is based on questionnaires, all reinterventions were registered regardless of the location where they took place. There is no bias because patients went to another caregiver or hospital. A strength of the study concerns the long follow-up period. This study is the first with such a long-term follow-up, which gives us a unique insight into the long-term effects and provides important data on uterine-preserving techniques.

In the last decade, the number of surgical interventions for POP and number of VH have decreased. Patients and their surgeons seem to be more hesitant to execute VH for POP, and this might indicate a shift towards more uterus-preserving procedures [5]. Since there is a lack of information about long-term follow-up, research like our cohort study is valuable for supporting patients in the decision-making process and improving future practice.

In conclusion, in our study there was no significant difference in subjective recurrence rates between the Manchester procedure and vaginal hysterectomy with low uterosacral ligament suspension. We did find that in the long term MF has a significantly shorter reintervention period. The small sample size and absolute difference in reoperation rate preclude a definite conclusion about non-inferiority, and future studies are needed. Meanwhile, shared decision making and careful deliberation of the pros and cons of all treatment options are needed in choosing the type of treatment for pelvic organ prolapse.

## Supplementary information

ESM 1 (DOCX 79 kb)
