# Supplementary material for: The modified Manchester Fothergill procedure compared with vaginal hysterectomy with low uterosacral ligament suspension in patients with pelvic organ prolapse: long-term outcome
Source: Int Urogynecol J. 2022 Jun 2;34(1):155–64. doi: 10.1007/s00192-022-05240-3 (PMC9160845; doi:10.1007/s00192-022-05240-3)
Supplement: Supplementary file 1 — (DOCX 79 kb) [file 192_2022_5240_MOESM1_ESM.docx]

# Appendices

## Appendix A: Questionnaire

**Vragenlijst MAFOVUE studie**De vergelijking tussen de Manchester Fothergill procedure en de vaginale baarmoeder verwijdering bij een bekkenbodemverzakking: de lange termijn resultaten

Allereerst willen we u namens het onderzoeksteam van harte bedanken voor uw tijd en moeite. Door uw inzet is het mogelijk om meer te weten te komen over verzakkingen na een verzakkingsoperatie dan wel een baarmoederverwijdering. Dit komt de patiënten voorlichting en mogelijk de behandeling ten goede. Voor u ligt de vragenlijst. Gemiddeld bent u ongeveer 10 minuten bezig met het invullen. Het eerste deel gaat over de verzakkingsoperatie dan wel baarmoederverwijdering die u in het verleden heeft gehad. Vervolgens enkele vragen over uw medische voorgeschiedenis. Als laatste een aantal vragen over bekkenbodemklachten.

Veel succes!

*Algemeen*

Lengte: ..........................................................

Gewicht: ..........................................................

Leeftijd: ………………………………………....

Datum van invullen: ..........................................................

Wat is uw huidige beroep of zijn uw werkzaamheden?

……………………………………………………………………………………….

*Hierna volgen enkele vragen over de verzakkingsoperatie en eventuele andere operaties. Gelieve het cijfer voor het antwoord dat voor u van toepassing is te omcirkelen. Probeert u alstublieft alle vragen te beantwoorden.*

*Voorgeschiedenis*

We hebben u benaderd omdat u in het verleden een operatie in verband met een verzakking heeft gehad.

1. Wat voor verzakkingsoperatie was dit?

1. Baarmoederverwijdering (vaginale uterusextirpatie)
2. Ophangen van de baarmoeder en verwijderen van baarmoedermond (Manchester Fothergill operatie)
3. Weet ik niet meer
4. anders, nl………………………………………..

2. Wanneer vond deze ingreep plaats? Jaar: ……....

3. Bent u destijds ook voor een andere verzakking dan de baarmoederverzakking geopereerd? (meerdere antwoorden mogelijk)

1. Nee
2. Ja, namelijk blaasverzakking (voorwand)
3. Ja, namelijk darmverzakking (achterwand)
4. Weet ik niet meer

4. Bent u na de operatie nogmaals behandeld voor een verzakking? (elke behandeling telt)

1. Nee (ga verder met vraag 11)
2. Ja

5. Zo ja, Wanneer bent u vanwege deze klachten
voor het eerst naar de huisarts of gynaecoloog gegaan? Jaar …….....

6. Er was toen sprake van een verzakking van de: (meerdere opties mogelijk)

1. Baarmoeder of vaginatop
2. Blaas
3. Darm
4. Weet ik niet

7. Kruis aan wat voor behandeling u heeft gehad (meerdere opties mogelijk)

1. Bekkenbodem fysiotherapie (ga door met vraag 11)
2. Ring (ga door met vraag 11)
3. Éénmalige operatie (ga door met vraag 8)
4. Meerdere operaties voor verzakking (ga door met vraag 8)

8. Wat voor operatie heeft u gehad voor de verzakking? (combinatie mogelijk)

1. Voor een blaasverzakking
2. Voor een darmverzakking
3. Voor een baarmoederverzakking
4. Voor een verzakking van de vaginatop
5. Weet ik niet

9. Wanneer vond deze ingreep plaats? Jaar …........

10. Indien er nogmaals een verzakkingoperatie nodig was, wat is er dan gedaan?

Operatie:…………………………………………….............. Jaar…………….

11. Heeft u een operatie voor urineverlies gehad? (TVT of TOT bandje)

1. Nee (ga verder met vraag 13)
2. Ja

12. Zo ja, wanneer vond deze ingreep plaats? Jaar …........

*De volgende vragen gaan over uw (eventuele) bevallingen. Omcirkel het antwoord dat voor u van toepassing is.*

13. Heeft u kinderen? 1 Ja 0 Nee

14. Hoe vaak bent u vaginaal bevallen? .......................... (aantal)

(indien 0 keer, ga door op volgende pagina)

15. Heeft u een keizersnede gehad? 1 Ja (......keer) 0 Nee

16. Heeft u een tang verlossing gehad? 1 Ja (......keer) 0 Nee

17. Heeft u een vacuüm cup verlossing gehad? 1 Ja (......keer) 0 Nee

18. Wanneer was uw laatste bevalling? Jaar …………………….

21. Hoe oud was u tijdens de eerste bevalling …………………………..

22. Bent u vaginaal bevallen van een kind met een geboortegewicht van meer dan 4000 gram?

1 Ja (……keer) 0 Nee

23. Hoe zou u uw algehele kwaliteit van leven gedurende de afgelopen week beoordelen?
*Omcirkel het getal dat het meest op u van toepassing is.*

1 2 3 4 5

Erg slecht Uitstekend

**Instructie:** Kruis bij iedere groep de zin aan die het best past bij uw eigen gezondheidstoestand vandaag.

24.a Mobiliteit

Ik heb geen problemen met lopen □ 1

Ik heb een enkel probleem met lopen □ 2

Ik heb enige problemen met lopen □ 3

Ik heb veel problemen met lopen □ 4

Ik ben bedlegerig □ 5

24.b Zelfzorg

Ik heb geen problemen om mijzelf te wassen of aan te kleden □ 1

Ik heb een enkel probleem om mijzelf te wassen of aan te kleden □ 2

Ik heb enige problemen om mijzelf te wassen of aan te kleden □ 3

Ik heb veel problemen om mijzelf te wassen of aan te kleden □ 4

Ik ben niet in staat om mijzelf te wassen of aan te kleden □ 5

24.c Dagelijkse activiteiten (b.v. werk, studie, huishouden, gezins- en vrije tijdsactiviteiten)

Ik heb geen problemen met mijn dagelijkse activiteiten □ 1

Ik heb een enkel probleem met mijn dagelijkse activiteiten □ 2

Ik heb enige problemen met mijn dagelijkse activiteiten □ 3

Ik heb veel problemen met mijn dagelijkse activiteiten □ 4

Ik ben niet in staat om mijn dagelijkse activiteiten uit te voeren □ 5

24.d Pijn / klachten

Ik heb geen pijn of andere klachten □ 1

Ik heb een beetje pijn of andere klachten □ 2

Ik heb matige pijn of andere klachten □ 3

Ik heb ernstige pijn of andere klachten □ 4

Ik ben zeer ernstige pijn of andere klachten □ 5

24.e Stemming

Ik ben niet angstig of somber □ 1

Ik ben een beetje angstig of somber □ 2

Ik ben matig angstig of somber □ 3

Ik ben erg angstig of somber □ 4

Ik ben heel erg angstig of somber □ 5

24.f
Om mensen te helpen bij het aangeven hoe goed of hoe slecht een gezondheidstoestand is, hebben we een meetschaal (te vergelijken met een thermometer) gemaakt. Op de meetschaal hieronder betekent de “100” de beste gezondheidstoestand die u zich kunt voorstellen, en de “0” de slechtste gezondheidstoestand die u zich kunt voorstellen.

We willen u nu vragen op de meetschaal aan te geven hoe goed of hoe slecht u eigen gezondheidstoestand vandaag is. Beweeg het blokje naar het cijfer om aan te geven hoe goed of hoe slecht volgens u uw gezondheidstoestand vandaag is. Kunt u aangeven hoe goed of hoe slecht uw gezondheidstoestand vandaag is?


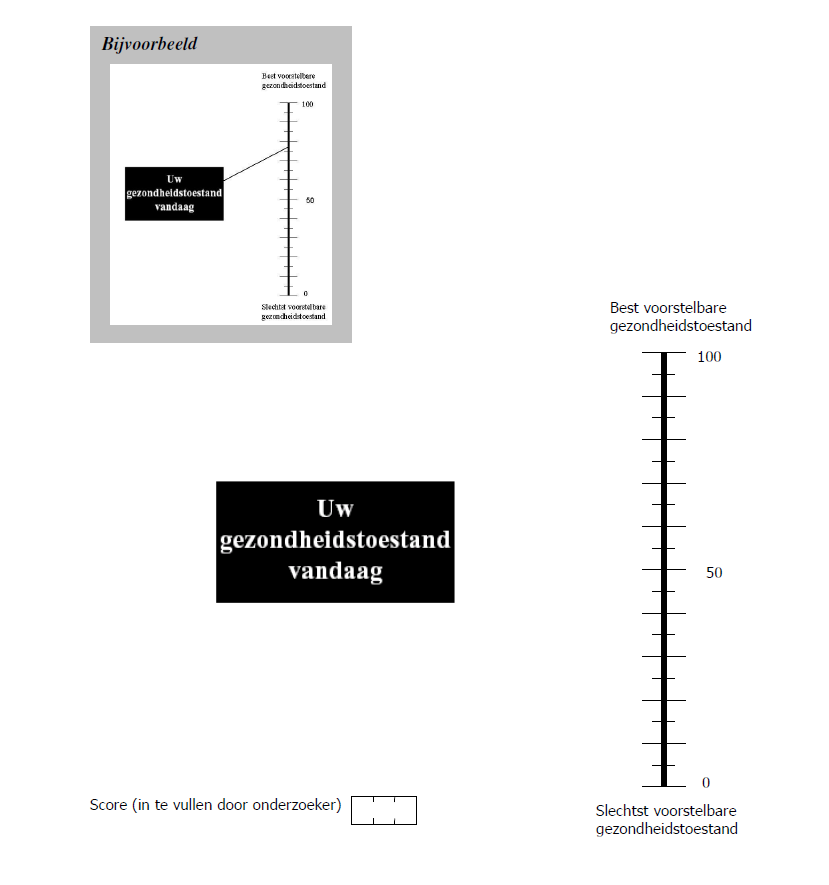


-------------------------------------------------------------------------------------------------------

Vrouwen met ongewenst urineverlies en / of een verzakking hebben aangegeven dat ze de volgende klachten hadden. Kunt u aangeven welke klachten u op dit moment ook heeft en hoeveel last u daar van heeft. Beantwoord s.v.p. alle vragen, ook als u geen klachten heeft.

--------------------------------------------------------------------------------------------------------

25. a. Vindt u dat u vaak moet plassen?

1 Ja 2 Nee (ga naar 25c.)

↓

b. Zo ja, hoeveel last heeft u hier van?

1 Helemaal niet 2 Een beetje 3 Redelijk wat 4 Heel erg

c. Hoe veel keer plast u gemiddeld per dag?: ..……..keer

26. a. Als u moet plassen voelt u dan altijd een sterke aandrang?

1 Ja 2 Nee (ga naar 27.)

↓

b. Zo ja, hoeveel last heeft u hier van?

1 Helemaal niet 2 Een beetje 3 Redelijk wat 4 Heel erg

27. a. Hebt u ongewenst urineverlies als u aandrang voelt om te plassen?

1 Ja 2 Nee (ga naar 28.)

↓

b. Zo ja, hoeveel last heeft u hier van?

1 Helemaal niet 2 Een beetje 3 Redelijk wat 4 Heel erg

c. Zo ja, hoe vaak verliest u ongewild urine?

1. dagelijks

2. paar keer per week

3. 1 keer per week

4. 1 keer per maand

5. 1 keer per jaar

28. a. Hebt u ongewenst urineverlies bij lichamelijke inspanning, hoesten of niezen?

1 Ja 2 Nee (ga naar 29.)

↓

b. Zo ja, hoeveel last heeft u hier van?

1 Helemaal niet 2 Een beetje 3 Redelijk wat 4 Heel erg

c. Zo ja, hoe vaak verliest u ongewild urine?

1. dagelijks

2. paar keer per week

3. 1 keer per week

4. 1 keer per maand

5. 1 keer per jaar

29. a. Hebt u moeite uw blaas leeg te plassen?

1 Ja 2 Nee (ga naar 30.)

↓

b. Zo ja, hoeveel last heeft u hier van?

1 Helemaal niet 2 Een beetje 3 Redelijk wat 4 Heel erg

30. a. Hebt u wel eens het gevoel dat de blaas na het plassen niet helemaal leeg is?

1 Ja 2 Nee (ga naar 31.)

↓

b. Zo ja, hoeveel last heeft u hier van?

1 Helemaal niet 2 Een beetje 3 Redelijk wat 4 Heel erg

31. a. Hebt u wel eens een drukkend gevoel onder in de buik?

1 Ja 2 Nee (ga naar 32.)

↓

b. Zo ja, hoeveel last heeft u hier van?

1 Helemaal niet 2 Een beetje 3 Redelijk wat 4 Heel erg

32. a. Hebt u wel eens pijn onder in de buik of in de schaamstreek?

1 Ja 2 Nee (ga naar 33.)

↓

b. Zo ja, hoeveel last heeft u hier van?

1 Helemaal niet 2 Een beetje 3 Redelijk wat 4 Heel erg

33. a. Hebt u wel eens het gevoel dat er iets uit de vagina stulpt?

1 Ja 2 Nee (ga naar 34.)

↓

b. Zo ja, hoeveel last heeft u hier van?

1 Helemaal niet 2 Een beetje 3 Redelijk wat 4 Heel erg

34. a. Hebt u wel eens gezien dat er iets uit de vagina stulpt?

1 Ja 2 Nee (ga naar 35.)

↓

b. Zo ja, hoeveel last heeft u hier van?

1 Helemaal niet 2 Een beetje 3 Redelijk wat 4 Heel erg

35. Hoe vaak hebt u het afgelopen jaar een blaasontsteking gehad?

1. Nooit

2. 1 keer

3. tussen de 2 en 4 keer

4. meer dan 4 keer

36. a. Moet u ‘s nachts meer dan 1 keer plassen?

1 Ja 2 Nee (ga naar 37.)

↓

b. Zo ja, hoeveel last heeft u hier van?

1 Helemaal niet 2 Een beetje 3 Redelijk wat 4 Heel erg

--------------------------------------------------------------------------------------------------------

De navolgende verschijnselen zijn beschreven door vrouwen met klachten van de stoelgang. Geeft u aan welke verschijnselen u tegenwoordig herkent en hoeveel last u daarvan heeft.

--------------------------------------------------------------------------------------------------------

37. a. Hebt u minder dan driemaal per week ontlasting?

1 Ja 2 Nee (ga naar 38.)

↓

b. Zo ja, hoeveel last heeft u hier van?

1 Helemaal niet 2 Een beetje 3 Redelijk wat 4 Heel erg

38. a. Moet u om ontlasting te krijgen in meer dan een kwart van de keren persen?

1 Ja 2 Nee (ga naar 39.)

↓

b. Zo ja, hoeveel last heeft u hier van?

1 Helemaal niet 2 Een beetje 3 Redelijk wat 4 Heel erg

39. a. Hebt u wel eens aandrang tot ontlasting terwijl er dan op het toilet geen ontlasting komt?

1 Ja 2 Nee (ga naar 40.)

↓

b. Zo ja, hoeveel last heeft u hier van?

1 Helemaal niet 2 Een beetje 3 Redelijk wat 4 Heel erg

40. a. Hebt u wel eens het gevoel dat er iets uit de anus hangt of er iets voor zit?

1 Ja 2 Nee (ga naar 41.)

↓

b. Zo ja, hoeveel last heeft u hier van?

1 Helemaal niet 2 Een beetje 3 Redelijk wat 4 Heel erg

41. a. Ervaart u pijn tijdens de aandrang tot ontlasting?

1 Ja 2 Nee (ga naar 42.)

↓

b. Zo ja, hoeveel last heeft u hier van?

1 Helemaal niet 2 Een beetje 3 Redelijk wat 4 Heel erg

42. a. Ervaart u pijn tijdens of vlak na de ontlasting?

1 Ja 2 Nee (ga naar 43.)

↓

b. Zo ja, hoeveel last heeft u hier van?

1 Helemaal niet 2 Een beetje 3 Redelijk wat 4 Heel erg

43. a. Verliest u wel eens dunne ontlasting zonder dat u daar controle over heeft?

1 Ja 2 Nee (ga naar 44.)

↓

b. Zo ja, hoeveel last heeft u hier van?

1 Helemaal niet 2 Een beetje 3 Redelijk wat 4 Heel erg

c. Hoe vaak komt het voor?

1. dagelijks

2. paar keer per week

3. 1 keer per week

4. 1 keer per maand

5. 1 keer per jaar

44. a. Verliest u wel eens vaste ontlasting zonder dat u daar controle over heeft?

1 Ja 2 Nee (ga naar 45.)

↓

b. Zo ja, hoeveel last heeft u hier van?

1 Helemaal niet 2 Een beetje 3 Redelijk wat 4 Heel erg

c. Hoe vaak komt het voor?

1. dagelijks

2. paar keer per week

3. 1 keer per week

4. 1 keer per maand

5. 1 keer per jaar

45. a. Verliest u wel eens windjes zonder dat u daar controle over heeft?

1 Ja 2 Nee (ga naar 46.)

↓

b. Zo ja, hoeveel last heeft u hier van?

1 Helemaal niet 2 Een beetje 3 Redelijk wat 4 Heel erg

c. Hoe vaak komt het voor?

1. dagelijks

2. paar keer per week

3. 1 keer per week

4. 1 keer per maand

5. 1 keer per jaar

46. a. Moet u wel eens via de schede mee drukken om ontlasting te krijgen?

1 Ja 2 Nee (ga naar 47.)

↓

b. Zo ja, hoeveel last heeft u hier van?

1 Helemaal niet 2 Een beetje 3 Redelijk wat 4 Heel erg

47. a. Moet u de ontlasting wel eens met de vingers via de anus verwijderen?

1 Ja 2 Nee (ga naar 48.)

↓

b. Zo ja, hoeveel last heeft u hier van?

1 Helemaal niet 2 Een beetje 3 Redelijk wat 4 Heel erg

Sommige vrouwen vinden dat ongewenst urineverlies en/of een verzakking en/of problemen met de ontlasting hun activiteiten, relaties en gevoelens kunnen beïnvloeden. De vragen in onderstaande lijst gaan over aspecten van uw leven die door uw probleem beïnvloed of veranderd kunnen zijn. Geef voor iedere vraag het antwoord aan dat het beste beschrijft hoe zeer uw activiteiten, relaties en gevoelens beïnvloed worden.

Hoeveel invloed heeft ongewenst urineverlies en/of verzakking en/of problemen met de ontlasting gehad op:

48. Uw vermogen om huishoudelijk werk te doen (koken, schoonmaken, wassen)

1 Helemaal niet 2 Een beetje 3 Redelijk wat 4 Heel erg

49. Uw vermogen om klein onderhoud of reparaties te verrichten in en om het huis

1 Helemaal niet 2 Een beetje 3 Redelijk wat 4 Heel erg

50. Boodschappen doen en winkelen

1 Helemaal niet 2 Een beetje 3 Redelijk wat 4 Heel erg

51. Reizen met auto of openbaar vervoer over een afstand van minder dan 20 minuten

1 Helemaal niet 2 Een beetje 3 Redelijk wat 4 Heel erg

52. Ergens naar toe gaan als u niet helemaal zeker weet of er daar toiletten zijn

1 Helemaal niet 2 Een beetje 3 Redelijk wat 4 Heel erg

53. Bezoek krijgen van vrienden en kennissen

1 Helemaal niet 2 Een beetje 3 Redelijk wat 4 Heel erg

54. Relaties met vrienden en kennissen

1 Helemaal niet 2 Een beetje 3 Redelijk wat 4 Heel erg

55. Vermogen om een seksuele relatie te hebben

1 Helemaal niet 2 Een beetje 3 Redelijk wat 4 Heel erg

56. Geestelijke / emotionele gezondheid

1 Helemaal niet 2 Een beetje 3 Redelijk wat 4 Heel erg

57. Wordt u in uw activiteiten beperkt door angst dat anderen u ruiken?

1 Helemaal niet 2 Een beetje 3 Redelijk wat 4 Heel erg

Hebt u als gevolg van uw probleem de volgende gevoelens?

58. Nervositeit of ongerustheid

1 Helemaal niet 2 Een beetje 3 Redelijk wat 4 Heel erg

59. Frustratie

1 Helemaal niet 2 Een beetje 3 Redelijk wat 4 Heel erg

60. Zich gegeneerd voelen

1 Helemaal niet 2 Een beetje 3 Redelijk wat 4 Heel erg

----------------------------------------------------------------------------------------------------------------

***De volgende vragen gaan over de seksualiteit.***

***Wanneer u bezwaar heeft tegen het invullen van deze vragen, mag u dit onderdeel van de vragenlijst (vraag 65 tot en met 69) ook overslaan. Natuurlijk stellen wij het op prijs wanneer u ze wel beantwoordt. Uw gegevens worden strikt vertrouwelijk behandeld.***

*Het is de bedoeling dat u bij het beantwoorden denkt aan de situatie van de afgelopen maand. Wilt U het voor u meest passende antwoord omcirkelen.*

----------------------------------------------------------------------------------------------------------------

61. a. Hebt u wel eens seksueel contact met uw partner? (Denk hierbij aan *alle vormen* van seksueel contact en niet alleen aan geslachtsgemeenschap)

1 Ja (beantwoord ook vraag b,c) 2 Nee (beantwoord ook vraag c)

b. Zo ja, hoe tevreden bent u daarover?

1 Helemaal niet 2 Een beetje 3 Redelijk wat 4 Heel erg

c. Zo nee, hoe vervelend vindt u dat?

1 Helemaal niet 2 Een beetje 3 Redelijk wat 4 Heel erg

62. Hoe vaak hebt u geslachtsgemeenschap?

1 Nooit

2 minder dan 1 keer per maand

3 1 tot 2 keer per maand

4 1 keer per week

5 meerdere keren per week

63. a. Verliest u wel eens urine tijdens de geslachtsgemeenschap?

1 Ja 2 Nee (ga naar 68.) 0 Niet van toepassing (geen seks)

↓

b. Zo ja, hoeveel last heeft u hier van?

1 Helemaal niet 2 Een beetje 3 Redelijk wat 4 Heel erg

64. a. Ervaart u pijn tijdens de geslachtsgemeenschap?

1 Ja 2 Nee (ga naar 69.) 0 niet van toepassing (geen seks)

↓

b. Zo ja, hoeveel last heeft u hier van?

1 Helemaal niet 2 Een beetje 3 Redelijk wat 4 Heel erg

65. a. Is de vagina zo nauw dat geslachtsgemeenschap daardoor niet mogelijk is?

1 Ja 2 Nee 0 niet van toepassing (geen seks)

↓

b. Zo ja, hoeveel last heeft u hier van?

1 Helemaal niet 2 Een beetje 3 Redelijk wat 4 Heel erg

66. Heeft u na de operatie nog andere klachten gekregen die voor de operatie niet aanwezig waren?

0 nee

1 ja

b. Zo ja, watvoor klachten? (meer dan één antwoord mogelijk)

1. pijn in de lies/bovenbeen,

2. een doof gevoel in de lies/bovenbeen,

3. een doof gevoel van de vagina,

4. het gevoel dat er iets trekt in de vagina,

5. een vreemd gevoel in de vagina.

6. anders namelijk;

………………………………………………………………………………………………

………………………………………………………………………………………………

………………………………………………………………………………………………

Mocht u opmerkingen of vragen hebben naar aanleiding van dit onderzoek, kunt u deze hier opschrijven.

………………………………………………………………………………………………

………………………………………………………………………………………………

………………………………………………………………………………………………

Heeft u op dit formulier **alle vragen** ingevuld?

Heeft u de toestemmingsverklaring getekend?

Gebruik de retourenvelop om de vragenlijst en getekende toestemmingsverklaring terug te sturen. Hier hoeft geen postzegel op.

Nogmaals hartelijk dank voor het invullen!
